# Broad North Atlantic distribution of a meiobenthic annelid – against all odds

**DOI:** 10.1038/s41598-019-51765-x

**Published:** 2019-10-29

**Authors:** Katrine Worsaae, Alexandra Kerbl, Áki Vang, Brett C. Gonzalez

**Affiliations:** 1 University of Copenhagen, Department of Biology, Marine Biological Section, Universitetsparken 4, 2100 Copenhagen Ø, Denmark; 2 Smithsonian Institution, National Museum of Natural History, Department of Invertebrate Zoology, MRC-163, P.O. BOX 37012, Washington, D.C. 20013 USA

**Keywords:** Taxonomy, Genetic variation, Biogeography, Marine biology

## Abstract

DNA barcoding and population genetic studies have revealed an unforeseen hidden diversity of cryptic species among microscopic marine benthos, otherwise exhibiting highly similar and simple morphologies. This has led to a paradigm shift, rejecting cosmopolitism of marine meiofauna until genetically proven and challenging the “Everything is Everywhere, but the environment selects” hypothesis that claims ubiquitous distribution of microscopic organisms. With phylogenetic and species delimitation analyses of worldwide genetic samples of the meiofaunal family Dinophilidae (Annelida) we here resolve three genera within the family and showcase an exceptionally broad, boreal, North Atlantic distribution of a single microscopic marine species with no obvious means of dispersal besides vicariance. With its endobenthic lifestyle, small size, limited migratory powers and lack of pelagic larvae, the broad distribution of *Dinophilus vorticoides* seems to constitute a “meiofaunal paradox”. This species feasts in the biofilm among sand grains, but also on macroalgae and ice within which it can likely survive long-distance rafting dispersal due to its varying lifecycle stages; eggs encapsulated in cocoons and dormant encystment stages. Though often neglected and possibly underestimated among marine microscopic species, dormancy may be a highly significant factor for explaining wide distribution patterns and a key to solving this meiofaunal paradox.

## Introduction

Within a single scoop of sand lies a diversity of microscopic metazoan organisms known as meiobenthos, defined as animals capable of passing a 0.5 mm mesh and being retained on a 42 μm mesh. These minute organisms belong either to ancestrally meiofaunal lineages (*e.g.,* Loricifera, Kinorhyncha, Gnathostomulida, Micrognathozoa, Gastrotricha, Rotifera, Nematoda, Tardigrada) or represent highly derived, meiofaunal descendants within macrofaunal groups (*e.g*. Arthropoda, Mollusca, Annelida, Echinodermata, Hemichordata)^1–3^. Whereas the local abundance, production and diversity of meiofauna may outnumber that of macrofauna^3–5^, the global diversity of meiofaunal animals has been deemed low and lacking biogeographical significance^6^.

With their simple and seemingly similar morphologies, many meiofaunal species appear to be morphologically static, previously regarded as being cosmopolitan across large geographical distances. Several explanatory hypotheses were put forward to explain these expansive distributions, including size^5^, dispersal models (*i.e*., stepping stone or long-distance transport) or that of vicariant events stemming from continental drift^7,8^. Subsequently, the “everything is everywhere, but the environment selects” (EiE) hypothesis (ubiquity theorem) became expanded to encompass meiofauna up to 2 mm^6^, although it was initially introduced to explain ubiquitous distribution of prokaryotes and unicellular eukaryotes. Uni- and multicellular organisms exhibit very different physiology, ecology and evolutionary histories, but an important shared attribute especially with limno-terrestrial meiofauna, is dormancy as an adaptation to temporary desiccation of habitats^8,9^. Multiple species of Nematoda, Arthropoda, Tardigrada, and Rotifera possess a desiccant-tolerant, dormant, long-term viable life stage such as thick-shelled resting eggs, cysts or cryptobiotic adults generally prone to airborne long-distance passive dispersal. Cosmopolitism is genetically proven for several of these species following the EIE hypothesis^8,9^.

Unlike limno-terrestrial meiofauna, marine meiobenthos usually persist under stable abiotic conditions and are generally lacking dispersal mechanisms such as pelagic larvae or dormant, long-term viable stages. With their small size, endobenthic lifestyle, limited migratory capabilities and lack of dispersal mechanisms, their supposed wide distribution indicated by morphological studies were a puzzle and ultimately coined as the “meiofauna paradox”^3,10,11^. Hence, this historical and contradictory framework has pushed the field forward, generating the need for combined detailed morphological and molecular analyses coupled with alternative modalities for testing meiofaunal patterns of distribution and dispersal^7,8,12,13^. Moreover, the necessity of considering the environmental differences and distinguishing between marine and limno-terrestrial meiofauna has become evident when testing hypothesis of meiofaunal distribution^8,12–15^.

Speaking against the EIE hypothesis and the meiofauna paradox, the diversity of marine meiofauna is generally understudied due to tedious sample processing and preservation techniques, coupled with lack of taxonomic expertise^3,5^. Aiding to resolve this hidden diversity, employment of advanced microscopy techniques and computational 3D reconstructions have provided a range of additional diagnostic anatomical traits in *e.g*., reproductive, nephridial, or sensory systems, demarcating the geographical span of many meiofaunal species^16,17^. Yet the most radical change in our comprehension of meiofauna diversity has occurred with DNA bar-coding and incorporation of molecular analyses into taxonomical studies^18,19^. Specifically, population genetics has revealed an unexpected high cryptic diversity among marine meiobenthic species, often limiting their geographical distribution^7,12,13,20–25^. This has caused a recent paradigm shift, now questioning the concept of marine meiofaunal cosmopolitism. Moreover, these findings suggest that marine meiofauna can reveal interesting biogeographical patterns, potentially more significant than marine macrofauna with pelagic larva and complex dispersal strategies.

However, some observations contradict original concepts coined by Sterrer^10^, and Danielopol and Wouters^26^ on infaunal meiobenthos being restricted to their very selective habitat: Representatives of nearly all marine meiofaunal groups, including meiofaunal annelids, arthropods and mollusks can routinely be found drifting or attached to eroded sand particles in the water column^4,15,27–29^ and have occasionally been found to travel distances of at least 10 kilometers^14^. While erosional tidal currents certainly aid their entry into the water column, ice, algae, and other marine or anthropogenic debris further facilitates their passive transport, potentially extending distances prior to resettlement^7,15,27,29^. Dormant, long-term viable stages are not common for marine meiofauna and their role in dispersal is therefore rarely investigated, but when encystment stages or resting eggs do exist, these may very likely enhance rafting and dispersal abilities. Regardless of the transport mechanism, the point of entry into the water column and often times the morphological and genetic identification remain unanswered, leading to biased and uncertain assumptions towards point source recruitment and necessitating more densely sampled population genetic studies of marine meiofauna.

Among meiofaunal groups, various annelid families are common throughout a broad range of environments and across vast geographical distances, yet densely sampled population genetic studies are rare^7,23^ and cosmopolitan species have not yet been genetically proven. The meiofaunal annelid family Dinophilidae has been recorded across the world for the last 150 years and is common in narrowly demarcated beach localities, often specific to either inter- or subtidal regions^30,31^. The family contains 18 valid species in two genera^31^, which lack significant annelid characters such as appendages, parapodia or chaetae and a free-swimming larval stage, but all show direct development of six, only internally discernible body segments and an external ventral ciliary tract used for gliding movement^31–34^. However, the various species exhibit three starkly different morphotypes that ultimately give way to three differing life cycles^31–38^: (i) species of *Trilobodrilus* Remane, 1925 are monomorphic with a life span of more than a year; from one moth old continuously producing interstitial offspring in spring and early summer, (ii) some species of *Dinophilus* O. Schmidt, 1848 are sexually highly dimorphic with short lived (~5 days) dwarf males and females sexually maturing within a month, reproducing continuously and surviving only a few months^31^, (iii) other species of *Dinophilus* are orange-colored, sexually monomorphic, and has a life span of nearly a year.

The orange-colored *Dinophilus vorticoides* O. Schmidt, 1848 (described from the Faroe Islands^32^) and *D. taeniatus* Harmer, 1889 (described from Plymouth, UK^33^) have dormant encystment stages notwithstanding desiccation but with increased tolerance to changing temperatures and salinities during summer/fall^35,36,39^. Unbeknown, the two species were synonymized as *D. taeniatus*^40^ and widely reported from the intertidal of the White Sea (Russia), Sweden, United Kingdom, and between the Faroe Islands and Greenland^31,40,41^. Despite *D. taeniatus* being only known from sediments or substrates, including algae or rocks, its seeming apparent capability of crossing large water bodies without pelagic larvae was largely ignored, owning up to the many conundrums of meiofauna.

Using widely accepted methods of molecular species delimitation, we here test the putative cross-Atlantic distribution of *D.vorticoides/D. taeniatus* whose encystment stage is possibly beneficial towards wider dispersal, sampling populations across the North Atlantic from the US East coast to the White Sea, with increased sampling densities throughout the Faroe Islands. Furthermore, we provide phylogenetic analyses of Dinophilidae, including members of each genus and across all life strategies from localities around the world (Suppl. Table 1), in order to test whether morphology and life cycles show systematic significance for classification of subgroups.

## Results

### Species delineation

Our molecular investigations into the cosmopolitan and morphologically identical ‘*Dinophilus taeniatus*/*vorticoides* clade’ showed that it consists of minimally two separate phylogenetic entities (Table 1), thereby reflecting the presence of the two originally established species^32,33^. One entity showed a surprising and exceptional broad boreal distribution across the North Atlantic from West Greenland to the White Sea, and is herein referred to as *D. vorticoides*, reviving the original name given to specimens collected from the Faroe Islands. The other entity, now (and so far) restricted to the Southwestern coasts of the United Kingdom, retains the name *D. taeniatus*.

**Table 1:** Species delimiting results for the *‘D. taeniatus/D. vorticoides’* clade using GMYC, PTP (m- and bPTP) calculated in BEAST, and ABGD methods for individual- and combined gene datasets.

	GMYC			mPTP	bPTP		ABGD
	ML entities (C.I.)	Likelihood ratio	*P*	Est. ent	Est. ent	Mean	Est. ent
COI	3 (2–11)	10.1525	0.0062*	2|*2*	2–12|*2–17*	3.95|*3.42*	2
CytB	3 (2–6)	16.4285	0.0003*	2|*9*	2–7|*18–24*	2.90|*22.91*	2
COI + CytB	7 (2–10)	12.3948	0.0020*	2|*2*	2–8|*2–6*	3.22|*3.07*	na
COI + 16S	5 (2–14)	10.6795	0.0048*	2|*1*	2–14|*13–27*	4.39|*22.78*	na
COI + 18S	7 (2–12)	9.7843	0.0075*	2|*2*	2–11|*2–24*	4.21|*4.28*	na
COI + 28S	3 (3–3)	15.6719	0.0004*	2|*2*	2–08|*2–17*	4.06|*4.08*	na
CytB + 16S	3 (2–5)	12.5169	0.0019*	2|*9*	2–10|*18–33*	3.56|*29.81*	na
CytB + 18S	3 (2–15)	14.4680	0.0007*	2|*5*	2–8|*13–33*	2.98|*29*.*27*	na
CytB + 28S	4 (2–5)	13.1257	0.0014*	2|*2*	2–13|*2–30*	3.19|*12.06*	na
COI + CytB + 16S	7 (4–14)	19.4123	0.0001*	2|*2*	3–10|*2–6*	3.59|*3.13*	na
COI + CytB + 18S	5 (2–10)	16.1894	0.0003*	2|*2*	2–9|*2–10*	3.38|*3.05*	na
COI + CytB + 28S	9 (2–10)	17.3548	0.0002*	2|*2*	2–13|*2–10*	3.42|*3*.*20*	na
COI + 16S + 18S	5 (2–12)	10.7169	0.0047*	2|*1*	2–12|*13–*2*8*	4.37|*22.62*	na
COI + 16S + 28S	3 (2–9)	10.4786	0.0053*	2|*2*	2–16|*2–16*	4.67|*4.31*	na
CytB + 16S + 18S	3 (2–6)	13.8879	0.0010*	2|*2*	2–7|*2–32*	3.16|*10.84*	na
CytB + 16S + 28S	3 (2–5)	11.8677	0.0026*	2|*2*	2–13|*2–33*	3.21|*6.80*	na
CytB + 18S + 28S	5 (5–10)	8.3728	0.0152*	2|*2*	2–24|*2–34*	6.03|*22.73*	na
COI + CytB + 16S + 18S	7 (3–14)	18.2361	0.0001*	2|*2*	3–9|*2–9*	3.47|*3.20*	na
COI + CytB + 16S + 28S	5 (2–13)	15.2130	0.0005*	2|*2*	2–12|*2–11*	3.61|*3.10*	na
COI + CytB + 18S + 28S	8 (2–13)	15.2821	0.0005*	2|*2*	2–10|*2–17*	3.46|*3.35*	na
COI + 16S + 18S + 28S	3 (2–11)	9.7713	0.0076*	2|*2*	2–17|*2–25*	4.45|*6.04*	na
CytB + 16S + 18S + 28S	3 (2–12)	13.1692	0.0014*	2|*2*	2–11|*2–34*	3.21|*15.14*	na
COI + CytB + 16S + 18S + 28S	8 (2–14)	15.1265	0.0005*	2|*2*	2–11|*2–14*	3.57|*3.44*	na

To test the strength of the bPTP and mPTP results, these analyses were additionally run in RaxML (values in italics). *p ≤ *0.05*, ML maximum likelihood, C.I. = confidence interval, Est. estimated, ent entities, na not applicable.

**Figure 1:**
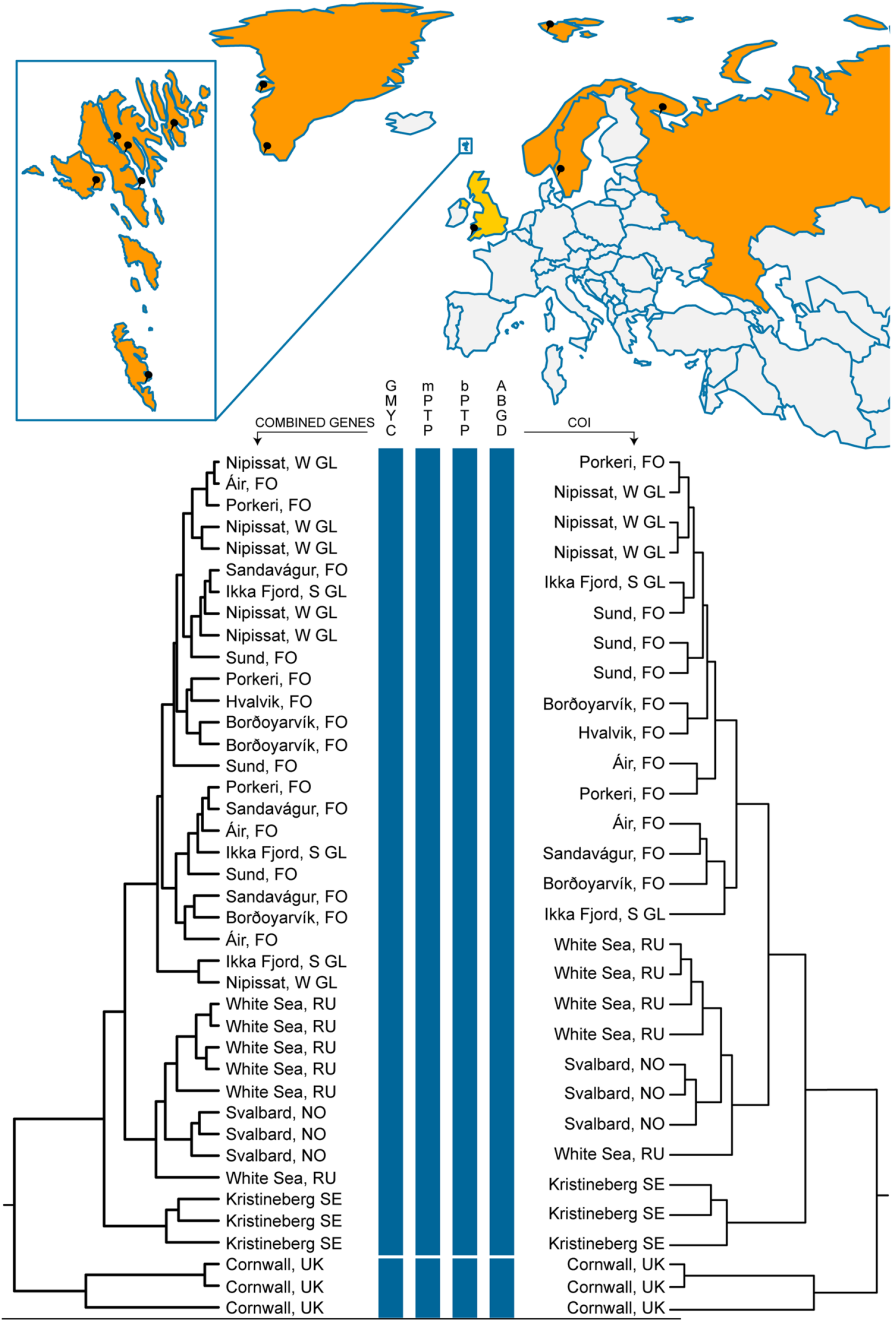
Distribution of collection localities for *Dinophilus vorticoides* Schmidt, 1848 and *Dinophilus taeniatus* Harmer, 1889 used in species delineations. Countries shaded in orange represent identified distributions of *D. vorticoides*, while countries in yellow represent *D. taeniatus*. Zoomed region indicates localities throughout the Faeroes Islands. Details pertaining to all pinned collections sites can be found in Suppl. Table 1. Figure bottom, ultrametric trees generated using combined five-gene dataset (left) and COI gene only dataset (right). Results of species delineations on right and left trees are indicated by vertical blue bars under each method. Breaks in vertical blue lines indicate clade based on conservative estimates with that method. Differences in number of taxa between ultrametric trees is due to removal of identical sequences prior to phylogenetic and delineation analyses.

Delineation results are based on comparisons of mitochondrial and ribosomal data from 39 specimens collected from 11 localities across the North Atlantic (Suppl. Table 1). Table 1 lists our delineation results across employed methods for identifying phylogenetic entities among single and concatenated gene datasets (18S rRNA, 28S rRNA and mitochondrial COI, CytB, 16S rRNA), and is illustrated in Fig. 1. We based our conclusions on phylogenetic entities revolving around the most conservative delineation estimates in common among the genetic markers and employed methods. GMYC analyses indicated the same two minimum entity estimates in all gene tree combinations with the exception of four instances where estimates suggested more than two identifiable entities (see Table 1). All GMYC analyses were statistically significant (*p* ≤ *0.05)*. Analyses using mPTP (BEAST trees) estimated two phylogenetic entities throughout all datasets tested (Table 1). When using bPTP (BEAST trees), two of the multiple gene tree combinations (COI + CytB + 16S and COI + CytB + 16S + 18S) recovered more than two entities. Comparative PTP analyses in RAxML likewise resulted in two phylogenetic entities for all larger compiled datasets as well as COI. Yet, our RAxML analyses failed to recover the minimum two entities in five (out of 23) of the smaller data sets (see Table 1). The variation in minimum entities recovered is most likely due to differences in tree building algorithms and how missing data is treated. ABGD analyses of the single gene datasets (COI and CytB) again recovered two distinct phylogenetic entities (Table 1).

Although we base our conclusions on the species delimitation analyses listed above, we also calculated genetic distances with Mega v7.0^42^ using the Kimura 2-parameter model with variation among sites modeled with a gamma distribution (shape parameter = 1). Positions containing missing data were eliminated. The number of base substitutions per site between sequences were highest for the sequenced COI fragment (645 bp) with comparable similarities among specimens of the 11 localities of *D. vorticoides* (97.2–100%) and among the single sampled population of *D. taeniatus* (97–99.7%), but with distances between these two sister clades being ten times higher (82.3–85.6% similarity) (Suppl. Table 2). Similarities were comparable for CytB (405 bp), ranging from 98.4–100% among *D. vorticoides* specimens, 96.6–98.5% among *D. taeniatus* specimens and 81.9–85.4% between these two sister species (Suppl. Table 3). For ribosomal genes, similarities were much higher within *D. vorticoides* and between the *D. vorticoides* and *D. taeniatus* specimens (16S rRNA (465 bp): 99.5–100% and 96–96.4%; 18S rRNA (1774 bp): 100% and 99.9%; 28S rRNA (1054 bp): 99.5–100% and 99.6–99.9%) (Suppl. Tables 4–6).

### Phylogenetic analyses

**Figure 2:**
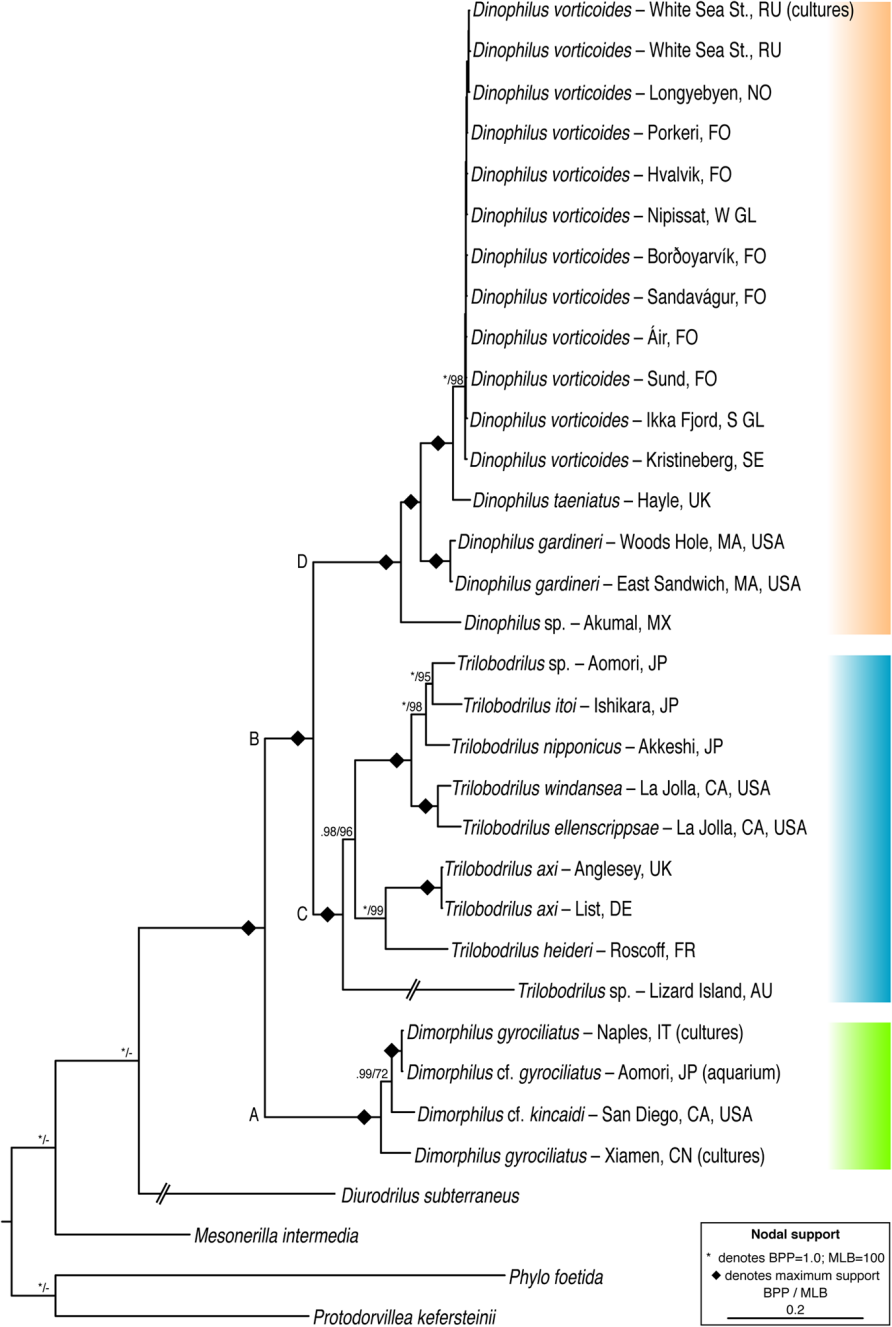
Phylogenetic relationships of Dinophilidae using combined gene analyses (18S rRNA, 28S rRNA, 16s rRNA, COI, CytB). Tree topology based on Bayesian analyses (BA) of combined gene datasets. Nodal support is indicated with both Bayesian posterior probabilities (BPP) and maximum likelihood bootstrapping (MLB). Only nodal support above BPP = 0.5 or MLB = 50 are indicated, those falling below this threshold are represented by as dash (−). Diamond (◆) shapes indicate maximum support: BPP = 1.0, MLB = 100. Color bars on right margin indicate the three recovered clades of Dinophilidae; orange, *Dinophilus;* blue, *Trilobodrilus;* green, *Dimorphilus* gen. nov.

Our phylogenetic investigation of Dinophilidae was conducted employing both Bayesian probabilities and Maximum likelihood analyses for all single gene datasets as well as our final concatenated five gene dataset (18S rDNA, 28S rDNA, 16S rDNA, COI, CytB). All analyses recovered Dinophilidae monophyletic, containing three well-supported clades found to represent differing morphologies and life cycles, but with *Dinophilus* being recovered paraphyletic (Fig. 2). Marked clades C, D, and A in Fig. 2 are here given generic ranks based on their morphology and phylogenetic status, with *Trilobodrilus* left unchanged, *Dinophilus* (sensu stricto) redefined, and a third clade erected and described herein as *Dimorphilus* gen. nov. This genus so far includes *D. gyrociliatus* (O. Schmidt, 1857) and *D. kincaidi* (Jones & Ferguson, 1957) (see diagnosis below). As per rules of the ICZN, the clade containing the nominal familial type species *D. vorticoides* will maintain the *Dinophilus* designation.

Dinophilidae was recovered fully supported (BPP = 1.0; MLB = 100) in all analyses and included two fully supported clades (BPP = 1.0; MLB = 100), one comprising taxa of the newly erected genus *Dimorphilus* (Clade A) and the other *Trilobodrilus* – *Dinophilus* (Clade B, Fig. 2). Clade B was recovered in all phylogenetic analyses and consists of separate clades for *Trilobodrilus* spp. (Clade C, Fig. 2) and *Dinophilus* spp. (Clade D, Fig. 2). The internal resolution within the *D. vorticoides* clade was not congruent among the analyses due to the low level of diversity among sequences.

The hyaline taxa of *Dimorphilus* (Clade A, Fig. 2) are characterized by having dwarf males, fast life cycles enabling year-round reproduction, and females with transverse rows of dorsal body ciliation. None of these taxa were wild caught, but sampled either from marine station aquaria or stemming from laboratory cultures (Suppl. Table 1). *Dimorphilus* cf. *gyrociliatus* cultures from Japan and *D. gyrociliatus* cultures from Italy formed a clade (BPP = 1.0; MLB = 100), which was found to nest with *D*. cf. *kincaidi* (BPP = 0.99; MLB = 72); the three of them forming a sister clade to *D. gyrociliatus*, Xiamen, China (Fig. 2).

Members of *Trilobodrilus* (Clade C, Fig. 2) are monomorphic, hyaline, lack dorsal body ciliation and their phylogenetic relationships seemingly correspond to geographic regions. *Trilobodrilus* sp. from Australia is sister to the clade *Trilobodrilus heideri* Remane, 1925 *– Trilobodrilus* sp. (BPP = 0.98; MLB = 96). Within this clade, subclades are formed corresponding to North Pacific and North Atlantic Oceans. *Trilobodrilus* from the North Pacific again comprise two subclades, one containing the West Pacific *T. windansea* Kerbl, Vereide, Gonzalez & Worsaae, 2018 and *T. ellenscrippsae* Kerbl, Vereide, Gonzalez & Worsaae, 2018 (BPP = 1.0; MLB = 100) from California, and the second containing the East Pacific *T. nipponicus* Uchida & Okuda, 1943 sister to *Trilobodrilus itoi* Kajihara, Ikoma, Yamasaki & Hiruta, 2015 *– Trilobodrilus* sp. (BPP = 1.0; MLB = 98) from Northern Japan. From the North Atlantic Ocean, *T. heideri* is sister to a fully supported clade of *T. axi* Westheide, 1967 from Germany and Wales (BPP = 1.0; MLB = 99).

The bright orange, monomorphic and dorsally ciliated members of Dinophilidae constitute Clade D (Fig. 2) or *Dinophilus* (sensu stricto) (BPP = 1.0; MLB = 96), which includes representatives from México (Mid-Atlantic) to the North Atlantic (Suppl. Table 1). *Dinophilus* sp. from México branches off as sister to the remaining North Atlantic species (*D. gardineri* Moore, 1900 (*D. taeniatus* – *D. vorticoides*)) (BPP = 1.0; MLB = 100). *Dinophilus gardineri* from East Sandwich and Woods Hole, USA form a fully supported sister relationship (BPP = 1.0; MLB = 100), again constituting a sister clade to the *D. taeniatus* – *D*. *vorticoides* clade (BPP = 1.0; MLB = 100). All specimens referred to as *D. vorticoides* form a comb-like subclade with nearly full support (BPP = 1.0; MLB = 98).

### Morphological examinations and taxonomic implications

**Figure 3:**
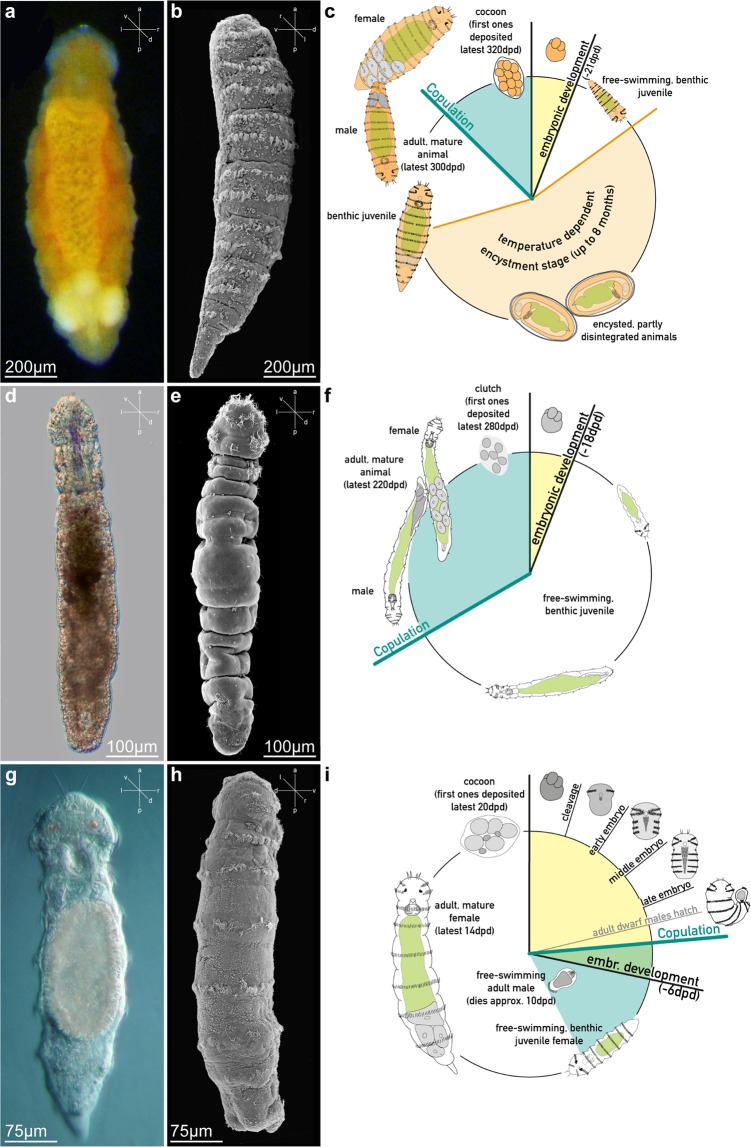
Morphology and life cycle of the three genera comprising Dinophilidae. (**a**–**c**) *Dinophilus*, exemplified by *D. vorticoides* Schmidt, 1848, collected from the Faroe Islands; (**d**–**f**) *Trilobodrilus*, exemplified by *T. axi* Westheide, 1967, collected from Sylt, Germany; and (**g**–**I**) *Dimorphilus* gen. nov., exemplified by *D. gyrociliatus* O. Schmidt, 1857, collected from cultures originating from Xiamen, China. a,d,g) Light microscopy images in dorsoventral view; b,e,h) scanning electron micrographs; b) in lateral view showing ventral ciliary tract and two segmental ciliary bands; (**e**) dorsal view showing lack of segmental ciliary bands; (**h**) in dorsolateral view showing one segmental ciliary band; c,f,i) schematic life cycle of the respective genus. The orientation of the microscopic images is indicated by small axis-schemes in each figure (a – anterior; d – dorsal; l – left; p – posterior; r – right; v – ventral).

Specimens, both live and fixed, belonging to the newly defined *Dinophilus* clade were examined using LM and SEM (Fig. 3) and all found to be strongly orange pigmented and sexually monomorphic. They have elongated cigar-shaped bodies with six indistinct trunk segments, a relatively broad mid-ventral ciliary band, and a prostomium with two pairs of anterior compound cilia and at least two incomplete transverse ciliary bands. *Dinophilus vorticoides* from West and South Greenland, Faroe Islands, Svalbard (Norway), Sweden, White Sea (Russia); *D. taeniatus* from Cornwall (United Kingdom); and *Dinophilus* sp. from Yucatán (México) show no obvious morphological differences and all possess two transverse ciliary bands per trunk on segments 1–5, with a dorsally incomplete transverse band on segment six. In contrast, the anterior prostomium and trunk of *D. gardineri* is densely ciliated with individual ciliary bands only distinguishable in the posterior trunk. These observations together with the phylogeny results in the following generic definitions.

Dinophilidae Macalister, 1876

***Dimorphilus*** gen. nov.

#### Type species

*Dimorphilus gyrociliatus* (O. Schmidt, 1857). Type locality: Naples, Italy (sampled from algae growing on the piers and rocky shore off Santa Lucia).

#### Diagnosis

Females with hyaline body with dark red pigmented kidney-shaped eyespots. Prostomium with two dorsally incomplete transverse ciliary bands. Six trunk segments, each with single transverse ciliary band. Strongly dimorphic dwarf males with minute round bodies, no distinct body regions or segmentation, presence of anterior, ventral and posterior ciliation and a muscular copulatory organ. Female life cycle completed within three weeks, males within a week.

#### Etymology

From Greek “dimorphos” (from *di-* ‘twice’ + *morphē* ‘form’), to account for the dimorphic sexes of the genus containing dwarf males, and from Greek “philos” (‘liking of’) in accordance with the similar ending of the type genus *Dinophilus*.

#### Remarks

Besides from *D. gyrociliatus*, only *D. kincaidi* is currently regarded a valid species within *Dimorphilus* based on morphology. Several species have been considered invalid due to poor descriptions and doubtful resemblance to dinophilid annelids^43^, often representing platyhelminths instead, *e.g., D. sphaerocephalus* Schmarda, 1861, *D. borealis* Diesing, 1862 and *D. rostratus* Schultz, 1902. However, species of *Dimorphilus* (especially *D. gyrociliatus*) have been reported from multiple disjunct localities around the world, some of which may represent genetically distinct species. For example, *Dimorphilus apatris* (Korschelt, 1882) and *D. conklini* (Nelson, 1907) were regarded junior synonyms to *D. gyrociliatus* due to morphological similarity^31^, but may in the future show to represent genetically separate species.

***Dinophilus*** (O. Schmidt, 1848)

#### Type species

*Dinophilus vorticoides* O. Schmidt, 1848. Type locality: Thorshavn, Faroe Islands, sampled from algae growing along the harbor.

#### Emended diagnosis

Strongly orange pigmented, monomorphic, with kidney-shaped red pigmented eyes. Prostomium, buccal region and trunk with transverse ciliary bands; antero-dorsal trunk ciliation dense or restricted to double segmental bands; posterior segments with one-two transverse ciliary bands each. Life cycle long with prolonged encystment stage.

### Remarks

Aside from *D. vorticoides, D. gardineri* (New England, USA) shows morphological differences and must be regarded a valid species. This study also proves *D. taeniatus* (Plymouth, UK) to represent a genetically different entity (opposing the previous synonymy with *D. vorticoides*^40^). *Dinophilus jägersteni* Jones and Ferguson, 1957 (US East Coast) shows some discrete differences in ciliation to *D. gardineri* and future genetic studies may prove its validity. Of the remaining putatively valid species of *Dinophilus*, *D. caudatus* (Fabricius, 1780): Levinson 1879–1880 and *D. metameroides* Hallez, 1879 were synonymized with *D. vorticoides* and *D. gigas* Weldon, 1886 was synonymized with *D. taeniatus*^31^, but these decisions may also have to be reevaluated by incorporating molecular data. *Dinophilus simplex* Verril, 1892 is invalid and was rejected by Ruebush^44^.

## Discussion

Molecular investigations of marine meiofauna generally argue against the ubiquity theorem (EiE)^20–25^, and the few meiofaunal annelids proposed to have broad amphi-Atlantic distributions have all been disproven cosmopolitans when investigated genetically^7,23^. Our recovered continuous boreal-Atlantic distribution of *D. vorticoides* is exceptional towards supporting the “Meiofauna paradox”, allowing us to now revisit controversial hypotheses, both on evolutionary stasis^10^ and EiE for marine meiofauna.

Broader distributions of meiofauna are now mainly attributed to contemporary events (*e.g.,* rafting, current, drift), integrating processes of dispersal, over that of supercontinent populations. However, while some limno-terrestrial meiofauna seems to fit the EIE hypothesis^8^ due to desiccant-tolerant stages capable of long-distance dispersal, desiccation tolerance in marine meiobenthos is very rare. Nonetheless, size and dormancy in various forms may still be a relevant trait for explaining wide distribution of marine meiobenthos, integrating processes of long-distance dispersal by means of megafauna, rafting, or even anthropogenic means of ballast water and sand^23,45–47^. Dinophilids constitute a highly derived evolutionary lineage within Annelida^48–51^ and show distinct and well supported clades and subclades (Fig. 2) contradicting evolutionary stasis. Moreover, *D. vorticoides* shows a mix of populations, especially among the Faroese and Greenland localities (see Fig. 1), whereby we attribute its broad distribution to a remarkable and previously inconceivable dispersal ability.

Dinophilidae, like other direct developing microscopic fauna, are considered to have low dispersal potential^10^ and the low genetic diversity among *D. vorticoides* populations recovered herein is counterintuitive given their direct development (=lacking pelagic larval stages) and their limited migratory abilities, being small and moving by ciliary action. On the other hand, *D. vorticoides* and *D. taeniatus* have an encystment stage extending from July to October/November in Sweden and UK; more northern populations have delayed onset and excystment extended sometimes into late winter. Both cysts and eggs encapsulated in gelatinous cocoons^35,36^ attach to substrates and are likely more tolerant to fluctuations in temperature and salinity than free moving adults^35^. Knowingly, all of Dinophilidae are described from interstitial sediments^31^, yet *D. vorticoides*, and likely other dinophilids, are not strictly bound by them^33,36^. Throughout our sampling, especially in the Faroe Islands, *D. vorticoides* was regularly found inhabiting filamentous algae and buoyant mollusk eggs, the later association not previously described. Seemingly, their willingness to selectively graze biofilms on drifting algae, buoyant invertebrate clutches, and potentially other marine debris (natural or anthropogenic) opens up their means of dispersal by ocean currents and winds^14^, especially in the egg or encystment phase of their life cycle. In the North Atlantic, floating algal clumps have long been associated as a type of microcosm or ‘micro-island’ that have the ability to transport and disperse macroscopic invertebrates, including larger annelids^45–47^. Although studies are limited and usually overlook micrometazoans, algal rafts are capable of traveling hundreds of kilometers, but the diversity of their faunal hitchhikers decreases with distance^45^. From a historical perspective, floating algae in European waters were responsible for seeding current algal communities throughout the Faroe Islands^46^ and is a widely accepted means by which the rocky shore flora and fauna of Eastern North America was established post glaciation^45^. Collections of *D. vorticoides* on filamentous algae are quite telling towards the likelihood of such a transport mechanism, and unlike most intertidal invertebrates, their internal copulation with later cocoon deposition and an encysted summer/fall (in the far north also including early winter) stage would potentially increase their tolerance during transportation, aiding towards successful colonization events and erection of small populations within short intervals.

There are however obstacles still obstructing our understanding of the processes shaping the current and seemingly cosmopolitan distribution in *D. vorticoides*, or with the same notion, what is restricting *D. taeniatus* from dispersing into the colder, northern localities of *D. vorticoides*? Our phylogenetic delineations suggest an absence of reproductive barriers among *D. vorticoides* populations, corroborated by low genetic diversities; however, identifying evidence of dispersal is far more convoluted. In Guil’s^9^ review of micrometazoans, it was discussed that dispersal may not always translate into gene flow, suggesting that numerous ecological and organismal conditions need to be met prior. One of these *a priori* conditions is that a continuous wave of individuals would be reaching each of the locations, however, our collection sites of *D. vorticoides* are often inundated with sea ice, altering normal colonization pathways. Furthermore, the polar ice coverage in the latest glacial period engulfed several of the current locations of *D. vorticoides*, now spanning relatively young oceanic areas. Interestingly, Jägersten^35^ observed *D. vorticoides* cysts encased in ice that continued to hatch upon thawing. Based on this indirect evidence of colonization along the retracting ice edge of the latest polar glacial coverage and the fact that our genetic distances within *D. vorticoides* has larger ranges than other meiofaunal investigations^7,23^, it would appear our distributions are heavily influenced by seasonal climatic events. Similarly, these events, including currents, may be restricting distributions of *D. taeniatus*, as northern Atlantic waters appear to not regularly mix with those surrounding the United Kingdom and/or *D. taeniatus* may not be able to tolerate the lower water temperatures in more northern waters. An interesting notion for future research is that genetically undetermined *D. vorticoides/D. taeniatus* populations have also been reported from more southern localities in e.g., Roscoff and Valencia, along the French and Spanish Atlantic coast, respectively^39^. It remains to be tested whether these populations represent the *D. taeniatus* species, which may hereby likewise have a broad but more southern distribution. While we are limited in our broad understanding of meiofaunal distribution in general, it appears that within Dinophilidae, especially *D. vorticoides*, represents a true cosmopolitan species, supporting the EiE (but the environment selects) hypothesis. While numerous questions still remain, our findings suggest that both historical and contemporary events are shaping distributions patters in meiofaunal annelids and likely to be group or even species specific. Our findings inevitably will provide a basis for future investigations whereby a more integrative approach focusing on connectivity may finally help in elucidating the meiofaunal paradox, *sensu stricto*.

Using most conservative estimates, species delineations of single and combined gene datasets have identified two significant *Dinophilus* clades throughout the North Atlantic that are more closely related to each other than to other *Dinophilus* taxa from the eastern United States and México. We hereby reject the previously proposed synonymy of *D. vorticoides* and *D. taeniatus*^31,40^, and accept the validity of both species, now recognizing *D. vorticoides* as the prolific species of the North Atlantic.

Dinophilidae is now demarked by three well-supported clades with distinct reproductive modes that eventually lead to the formation of gelatinous cocoons (in *Dinophilus* and *Dimorphilus*) or clutches (in *Trilobodrilus*) that house yolky eggs. These cocoons or clutches are deposited in favorable habitats, including sediments, on algae, and along rocks or pilings, giving rise to directly developed free-swimming, but benthic, juveniles (Fig. 3). Systematically, these clades include *Dimorphilus* gen. nov., representing the smallest dinophilids (females ≤ 1.3 mm), displaying strong sexual dimorphism with completely hyaline dwarf males and females with a single ciliary band per segment and a rapid life cycle; *Trilobodrilus* (≤2.0 mm), being hyaline with a distinctive trilobed prostomium and limited ciliation on the trunk^30,31,52^; and *Dinophilus*, being the largest dinophilids (≤3 mm), with easily recognizable orange-red pigmentation and two transverse ciliary bands on each trunk segment or a more random and occasionally denser distribution of ciliary tufts^34,35,40,44^. From a strictly morphological standpoint, *Dimorphilus* (*e.g., D. gyrociliatus*) shares several diagnostic morphological features of *Dinophilus* (*e.g., D. taeniatus*), so their previously proposed affinity had never come into question.

By incorporating years of meiofaunal collections, our phylogenetic analyses of Dinophilidae now provides clear support of separate evolutionary pathways between monomorphic and dimorphic taxa, promoting a common monomorphic ancestor for the clade *Trilobodrilus – Dinophilus*. Unfortunately, we cannot provide further insight whether the traits of the paedomorphic *Dimorphilus* are more closely representing the ancestor of all Dinophilidae or present a series of derived characters. Prior to this study, the most extensive molecular phylogenies within Dinophilidae were focusing on the systematics within *Trilobodrilus*^1,53^, and regrettably, these phylogenies only included a single *Dinophilus* species (syn. as *Dimorphilus*). While Kajihara *et al*.^53^ did include *Dinophilus* sp. from Lizard Island (Australia) (previously published by Worsaae & Rouse^54^), it had initially been incorrectly identified, thus preventing the discovery of a paraphyletic *Dinophilus*. This specimen was herein reexamined by means of LM and reidentified as *Trilobodrilus* sp.

In addition to published results, the composition of *Dimorphilus*, while fully supported (Fig. 2), is confusing since cultures once maintained by Bertil Åkesson of *D. gyrociliatus* collected in China, Italy, etc. have since been dispersed, making it unfeasible to trace if mixing of cultures has occurred, or which genetic sample stem from the type locality. While this was not the focus of the study, it appears that multiple independent lineages of *D. gyrociliatus* are being used throughout the literature (see David & Halanych^55^), and our integration of sequences from both marine aquaria and laboratory cultures suggest that a larger and unidentified diversity is currently present within *Dimorphilus*.

## Methods

In order to determine the extent of the distribution of *D. taeniatus*, samples were collected from coastal areas throughout the Faroe Islands, Greenland, México, Sweden, Norway, and the White Sea, Russia. Specimens were also obtained from the type locality of *D. vorticoides* in the Faroe Islands and for *D. taeniatus* from the United Kingdom in order to determine the validity and phylogenetic extent of these species.

### Collection and examination

Interstitial members of Dinophilidae were extracted from fine sand and coral rubble^56^ collected from the intertidal zone to 20 meters depth. Epibiont and epibenthic specimens were collected by hand from rocks, algae or pilings.

Samples for molecular analyses were preserved in 96–100% ethanol while vouchers and specimens for morphological examination were fixed in either 3% glutaraldehyde or 2–4% paraformaldehyde as previously outlined^17,34,52,54^.

Light microscopy (LM) was used to examine newly acquired live material using an Olympus IX70 inverted microscope mounted with an Olympus DP73 digital camera. Detailed morphological examinations were made using a JEOL JSM-63335F field emission scanning electron microscope (SEM) at the Natural History Museum of Denmark, University of Copenhagen. Specimens were prepared for SEM following previously published protocols that included fixation in glutaraldehyde, postfixation in osmium tetroxide, and dehydration by an ascending ethanol series^30,54^. Prior to imaging, all specimens were critical-point dried, mounted on aluminum stubs, and sputter-coated with platinum/palladium.

### Molecular laboratory methods

Evolutionary relationships within Dinophilidae were examined using the ribosomal markers 18S rRNA, 28S rRNA and 16S rRNA, as well as the mitochondrial markers cytochrome *c* oxidase subunit I (COI) and cytochrome B (CytB). The combinations of conserved and fast evolving genes were selected to resolve inter- and intraspecific relationships among and between the dinophilid genera. Additionally, given that the three selected mitochondrial markers are fast evolving, they were also selected to resolve population level dynamics within the ‘*D. taeniatus*/*D. vorticoides* clade’.

Total genomic DNA was obtained from individual dinophilid specimens using the Qiagen DNeasy Tissue & Blood Kit (Qiagen Inc., Valencia, CA, USA) following the manufactures protocol. DNA was extracted from at least three separate individuals from each of the collection localities of ‘*D. taeniatus*/*D. vorticoides*’.

Samples were prepared for polymerase chain reactions (PCR), sequenced and aligned according to methods previously outlined^30^.

Generated sequences were deposited in GenBank® and their accession numbers can be found in Suppl. Table 1.

### Dataset assembly

To understand the relationships among the genera of Dinophilidae and identify the closest relative of the *D. vorticoides*/*D. taeniatus* clade, phylogenetic reconstructions for Dinophilidae were performed using gene data from only a single representative from each collection locality as well as any already deposited information available on GenBank. For species delineations within the ‘*D.vorticoides*/*D. taeniatus* clade’, identical sequences were first identified using pairwise distances in Bioedit^57^ and subsequently removed to avoid inclusion of redundant information.

Due to the fact that the position of Dinophilidae is still highly debated and unresolved, outgroup selection was based on recovered sister group relationships from recent phylogenomic investigations^48,49^. Outgroup representatives included *Mesonerilla intermedia* Wilke, 1953 (Nerillidae), *Diurodrilus subterraneus* Remane, 1934 (Diurodrilidae), *Protodorvillea kefersteini* (McIntosh, 1869) (Dorvilleidae), and *Phylo foetida* (Claparède, 1868) (Orbiniidae).

Sequences were aligned using the MAFFT online platform^58^. Individual gene datasets were aligned under the L-INS-I interactive refinement method^59^. Datasets for 18S rRNA and 28S rRNA were aligned with the ‘nwildcard’ option selected, as this does not designate missing data as gaps. Alignments of COI and CytB were trivial, however, both datasets were aligned to check for directionality. Protein coding genes COI and CytB were checked for stop codons prior to phylogenetic analyses using Mesquite v.3.51^60^. Individual gene datasets were concatenated using Sequence Matrix^61^.

### Phylogenetic analyses

Phylogenetic reconstructions were performed on individual gene datasets, as well as concatenated gene datasets, using both maximum likelihood (ML) and Bayesian methods.

Maximum likelihood analyses were performed in RAxML v.7.2.8^62^ as implemented on the CIPRES Science Gateway^63^. Given that RAxML only implements general time reversible (GTR) models of sequence evolution for amino acids, a GTR model with corrections for discrete gamma distribution (GTR + Γ) was specified for individual gene and concatenated gene datasets. Non-parametric bootstrapping with 1,000 replicates was used to generate nodal support estimations^64^.

Bayesian analyses (BA) were performed using MrBayes v.3.2.6^65^ as implemented on the CIPRES Science Gateway^63^. Prior to analyses, jModelTest^66^ was used on each individual gene dataset (18S rRNA, 28S rRNA, 16 s rRNA, COI, CytB) to evaluate their optimal evolutionary model as estimated by the corrected Akaike information criterion (AICc). A GTR model with gamma distribution and a proportion of invariable sites (GTR + I + Γ) was shown to be the best estimate for 18S rRNA, COI, and CytB, while 28S rRNA and 16S rRNA were selected for a GTR + Γ model. Both individual and concatenated datasets were run with two independent analyses using four chains (three heated, one cold). Generation sampling was set to 30 million, sampling every 1000 generations. Burnin was set to 10 million generations. Majority-rule consensus trees (50%), posterior probabilities, and branch lengths were constructed using the remaining trees after burnin. Convergence of all MCMC runs were verified using TRACER v.1.6.0^67^.

### Species delimitation

We employed an integrative taxonomic approach, including morphological (LM and SEM) and DNA taxonomy to determine if *D. taeniatus* represented a single species throughout the sampled localities, or at the opposite extreme, if each population could be considered separate and an independently evolving entity. Three methods widely employed in DNA taxonomy were used^18^, including the generalized mixed Yule-coalescent (GMYC) model^68^, the Poisson tree process (PTP), including multi-rate (mPTP)^69^ and Bayesian implementation (bPTP)^70^, and the Automated Barcode Gap Discovery (ABGD)^71^. Outgroups were removed prior to implementation of the beforementioned methods.

Methods of GMYC, mPTP, and bPTP utilized ultrametric trees generated using Bayesian Inference in BEAST (see below). Since BEAST generates ultrametric trees without smoothing (no data loss), all analyses incorporated BEAST trees to maintain consistency across methods used when generating tree topologies. Yet, to test the strength of our mPTP and bPTP delineations, these analyses were additionally run with methods of maximum likelihood, generating trees in RAxML v.7.2.8^62^ (following methods listed above). Analyses using GMYC were performed in R v.3.5.1 (R Core Team, 2014) using the package SPLITS v.1.0-19^72^ on phylogenies obtained from individual and combined datasets. PTP analyses were carried out on the mPTP online server (http://mptp.h-its.org) and the bPTP online server (http://species.h-its.org). No changeable settings are present on the mPTP online server, however, bPTP analyses were run using 10^4^ MCMC generations with a burning of 0.1. Individual COI and CytB datasets were uploaded on the ABGD online platform (http://wwwabi.snv.jussieu.fr/public/abgd/abgdweb.html) and were analyzed using preset parameters^70^.

Ultrametric trees were generated using Bayesian Inference in BEAST v.1.8.4^73^ as implemented on the CIPRES Science Gateway^63^. BEAUTi (Bayesian Evolutionary Analysis Utility) v.1.8.4 generated xml files for all BEAST runs. Independent BEAST runs were created for individual and well as combined and partitioned datasets. Tree priors for all analyses were selected under a Coalescent Process with constant population size. Nucleotide substitution models were estimated by the corrected Akaike information criterion (AICc) using jModelTest^66^. Both 18S rRNA and COI were selected for a generalized time reversible model with a proportion of invariable sites (GTR + I), 28S rRNA and 16S rRNA were selected for GTR with gamma distribution (GTR + Γ), and CytB was selected for Hasegawa, Kishino, and Yano model with gamma distribution (HKY + Γ). All datasets had independent MCMC analyses with 10^8^ generations and trees were sampled every 10000 generations. TRACER v.1.6.0^67^ was used to verify convergence of all MCMC runs. A maximum clade credibility (MCC) consensus tree was obtained for each BEAST dataset in TreeAnnotator v.1.8.4 after annotating the remaining 9001 trees after burnin.

## Supplementary information

Supplementary material
