# Author Correction: Broad North Atlantic distribution of a meiobenthic annelid – against all odds

**DOI:** 10.1038/s41598-021-86691-4

**Published:** 2021-03-31

**Authors:** Katrine Worsaae, Alexandra Kerbl, Áki Vang, Brett C. Gonzalez

**Affiliations:** 1 University of Copenhagen, Department of Biology, Marine Biological Section, Universitetsparken 4, 2100 Copenhagen Ø, Denmark; 2 Smithsonian Institution, National Museum of Natural History, Department of Invertebrate Zoology, MRC-163, P.O. BOX 37012, Washington, D.C 20013 USA

Correction to: *Scientific Reports* 10.1038/s41598-019-51765-x, published online 29 October 2019

The original version of this Article did not include evidence of registration in ZooBank within the work itself, as required by Article 8.5.3 of the International Code of Zoological Nomenclature^1^. Therefore, the details of the newly proposed genus name *Dimorphilus* and a description of the Nomenclatural Acts were not available at the time of publication.

This published work and the Nomenclatural Acts it contains have been registered in ZooBank with the LSID urn:lsid:zoobank.org:pub:956E521E-E689-4F92-848C-D8868200A483 for this publication and urn:lsid:zoobank.org:act:3E3FDE0E-3A43-4320-9776-6E4E4ACF78C0 for the genus.

The original Article has been corrected.
