# Supplementary material for: Broad North Atlantic distribution of a meiobenthic annelid – against all odds
Source: Sci Rep. 2019 Oct 29;9:15497. doi: 10.1038/s41598-019-51765-x (PMC6820731; doi:10.1038/s41598-019-51765-x)
Supplement: Supplementary file 1 — Supplementary material [file 41598_2019_51765_MOESM1_ESM.pdf]

Broad North Atlantic distribution in a meiobenthic annelid – against all odds

Katrine Worsaae[1*], Alexandra Kerbl[1], Áki Vang[1], Brett C. Gonzalez[1,2*]

1. University of Copenhagen, Department of Biology, Marine Biological Section, Universitetsparken 4, 2100 Copenhagen Ø, Denmark
2. Smithsonian Institution, National Museum of Natural History, Department of Invertebrate Zoology, MRC-163, P.O. BOX 37012, Washington, D.C. 20013, USA

* kworsaae@bio.ku.dk; gonzalezb@si.edu.

Supplementary Table 1. Details of specimens and sequences used in this study. Identical sequences of *D. vorticoides*/*D. taeniatus* specimens were excluded from species delimitation analyses; phylogenetic analyses of the entire family included one representative from each locality.

Taxon	Country	Locality	Coordinates	Extraction ID#	18S rDNA	28S rDNA	16S rDNA	COI	CyB	Phylo-genetic analyses	Delimi-tation analyses
Trilobodrilus axi	United Kingdom	Anglesey, Wales	53°18'09"N 04°11'07"W	KW747	MK894523	MK894459	MK894492		MK896764	+	
Trilobodrilus axi	Germany	List, Sylt	55°01'38"N 08°25'57"E	KW760	MK894524	MK894460	MK894491	MK896709	MK896768	+	
Trilobodrilus heideri	France	Trezen ar Skoden, Roscoff	48°45′55″N 04°06′45″W	KW297	MK894522	MK894461	MK894496	MK896666	MK896717		
Trilobodrilus heideri	France	Trezen ar Skoden, Roscoff	48°45′55″N 04°06′45″W	KW592	MK894521	MK894462	MK894497	MK896699	MK896755	+	
Trilobodrilus sp.	Japan	Junahama Beach, East Mutsu Bay, Aomori	41°04'30"N 141°11'01"E	KW745	MK894520	MK894549	MK894495	MK896705	MK896763	+	
Trilobodrilus itoi[1]	Japan	Ishikari Beach, Ishikari, Hokkaido			AB924372	AB924373		AB924371		+	
Trilobodrilus nipponicus[1]	Japan	Cape Aikappu, Akkeshi, Hokkaido			LC009446	LC009447		LC009445		+	
Trilobodrilus windensea[2]	United States of America	Windansea Beach, La Jolla, CA	32°49′46″N 117°16′51″W	KW161	MG588089	MG588091	MK894493	MG588093	MG588095	+	
Trilobodrilus ellenscrippsae[2]	United States of America	La Jolla, CA	32°51′03″N 117°16′20″W	KW167	MG588090	MG588092	MK894494	MG588094	MG588096	+	
Trilobodrilus sp.	Australia	Lagoon, Lizard Island	14°41'00"S 145°27'20"E	KW761	MK894544	MK894480			MK896769	+	
Dinophilus vorticoides	Faroe Islands	Borðoyarvík, Norðoyar	62°12'55"N 06°34'08"W	KW571a	MK894526	MK894474	MK894500	MK896668	MK896719	+	+
Dinophilus vorticoides	Faroe Islands	Borðoyarvík, Norðoyar	62°12'55"N 06°34'08"W	KW571b				MK896669	MK896720		
Dinophilus vorticoides	Faroe Islands	Borðoyarvík, Norðoyar	62°12'55"N 06°34'08"W	KW571c				MK896670	MK896721		+

Dinophilus vorticoides	Faroe Islands	Hvalvik, Streymoyar	62°11'41"N 07°00'13"W	KW185	MK894525	MK894463	MK894498	MK896664	MK896716	+	+
Dinophilus vorticoides	Faroe Islands	Áir, Streymoyar	62°10'21"N 06°58'14"W	KW573a	MK894532	MK894471	MK894501	MK896674	MK896725	+	+
Dinophilus vorticoides	Faroe Islands	Áir, Streymoyar	62°10'21"N 06°58'14"W	KW573b				MK896675	MK896726		+
Dinophilus vorticoides	Faroe Islands	Áir, Streymoyar	62°10'21"N 06°58'14"W	KW573c				MK896676	MK896727		
Dinophilus vorticoides	Faroe Islands	Sund, Streymoyar	62°02'53"N 06°49'53"W	KW581	MK894538	MK894470	MK894502	MK896691	MK896747	+	+
Dinophilus vorticoides	Faroe Islands	Sund, Streymoyar	62°02'53"N 06°49'53"W	KW582				MK896692	MK896748		+
Dinophilus vorticoides	Faroe Islands	Sund, Streymoyar	62°02'53"N 06°49'53"W	KW589				MK896693	MK896749		+
Dinophilus vorticoides	Faroe Islands	Porkeri, Suðuroy	61°28'49"N 06°44'46"W	KW642a	MK894529	MK894468	MK894505	MK896700	MK896757	+	+
Dinophilus vorticoides	Faroe Islands	Porkeri, Suðuroy	61°28'49"N 06°44‘46"W	KW642b				MK896701	MK896758		+
Dinophilus vorticoides	Faroe Islands	Porkeri, Suðuroy	61°28'49"N 06°44'46"W	KW642c				MK896702	MK896759		
Dinophilus vorticoides	Faroe Islands	Sandavágur, Vágar	62°03'02"N 07°09'07"W	KW572a		MK894473	MK894499	MK896671	MK896722	+	+
Dinophilus vorticoides	Faroe Islands	Sandavágur, Vágar	62°03'02"N 07°09'07"W	KW572b	MK894535			MK896672	MK896723		
Dinophilus vorticoides	Faroe Islands	Sandavágur, Vágar	62°03'02"N 07°09'07"W	KW572c				MK896673	MK896724		
Dinophilus vorticoides	Greenland	Nipissat, Disko Island	69°25'56"N 54°10'37"W	KW574a					MK896728		+
Dinophilus vorticoides	Greenland	Nipissat, Disko Island	69°25'56"N 54°10'37"W	KW574b			MK894510	MK896677	MK896729		+
Dinophilus vorticoides	Greenland	Nipissat, Disko Island	69°25'56"N 54°10'37"W	KW574D					MK896730		+
Dinophilus vorticoides	Greenland	Nipissat, Disko Island	69°25'56"N 54°10'37"W	KW574f				MK896678	MK896731		+
Dinophilus vorticoides	Greenland	Ikka Fjord	61°11'28"N 48°01'40"W	KW590a	MK894531	MK894472	MK894511	MK896694	MK896750	+	+
Dinophilus vorticoides	Greenland	Ikka Fjord	61°11'28"N 48°01'40"W	KW590b				MK896695	MK896751		
Dinophilus vorticoides	Greenland	Ikka Fjord	61°11'28"N 48°01'40"W	KW590c				MK896696	MK896752		+
Dinophilus vorticoides	Greenland	Nipissat, Disko Island	69°25'56"N 54°10'37"W	KW763	MK894533	MK894464	MK894508	MK896710	MK896770	+	
Dinophilus vorticoides	Greenland	Nipissat, Disko Island	69°25'56"N 54°10'37"W	KW764	MK894537		MK894509	MK896711	MK896771		+
Dinophilus vorticoides	Norway	Longyearbyen, Svalbard	78°13'35"N 15°38'20"E	KW576a	MK894527	MK894467	MK894507	MK896682	MK896735	+	+
Dinophilus vorticoides	Norway	Longyearbyen, Svalbard	78°13'35"N 15°38'20"E	KW576b				MK896683	MK896736		+
Dinophilus vorticoides	Norway	Longyearbyen, Svalbard	78°13'35"N 15°38'20"E	KW576c				MK896684	MK896737		+

Dinophilus vorticoides	Sweden	Kristineberg	58°15'60"N 11°27'07"E	KW575a	MK894530	MK894475	MK894506	MK896679	MK896732	+	+
Dinophilus vorticoides	Sweden	Kristineberg	58°15'60"N 11°27'07"E	KW575b				MK896680	MK896733		+
Dinophilus vorticoides	Sweden	Kristineberg	58°15'60"N 11°27'07"E	KW575c				MK896681	MK896734		+
Dinophilus vorticoides	Russia	White Sea Biological Station, Velikaja Salma Bay, White Sea (culture)	66°33'13"N 33°06'16"E	KW510	MK894534	MK894465	MK894512	MK896667	MK896718	+	
Dinophilus vorticoides	Russia	White Sea (culture)	66°33'13"N 33°06'16"E	KW578a	MK894528	MK894476	MK894513	MK896688	MK896741	+	+
Dinophilus vorticoides	Russia	White Sea (culture)	66°33'13"N 33°06'16"E	KW578b				MK896689	MK896742		+
Dinophilus vorticoides	Russia	White Sea (culture)	66°33'13"N 33°06'16"E	KW578c				MK896690	MK896743		+
Dinophilus vorticoides	Russia	White Sea (culture)	66°33'13"N 33°06'16"E	KW768	MK894540	MK894466	MK894503	MK896712	MK896772		+
Dinophilus vorticoides	Russia	White Sea (culture)	66°33'13"N 33°06'16"E	KW769	MK894536		MK894504	MK896713	MK896773		+
Dinophilus taeniatus	United Kingdom	Carnsew pool, Hayle, Cornwall	50°11'02"N 05°25'40"W	KW756			MK894514	MK896706	MK896765		+
Dinophilus taeniatus	United Kingdom	Carnsew pool, Hayle, Cornwall	50°11'02"N 05°25'40"W	KW757	MK894539	MK894469	MK894516	MK896707	MK896766	+	+
Dinophilus taeniatus	United Kingdom	Carnsew pool, Hayle, Cornwall	50°11'02"N 05°25'40"W	KW758			MK894515	MK896708	MK896767		+
Dinophilus gardineri	United States of America	Eel pond, Woods Hole, MA	41°31'34"N 70°40'13"W	KW690	MK894541	MK894477	MK894517	MK896703	MK896760	+	
Dinophilus gardineri	United States of America	Cape Cod Bay, East Sandwich, MA	41°43'26"N 70°20'47"W	KW692	MK894542	MK894478	MK894518	MK896704	MK896761	+	
Dinophilus sp.	México	Mesoamerican Reef, Akumal	20°24'12"N 87°17'56"W	KW591a	MK894543	MK894479	MK894519	MK896697	MK896753	+	
Dinophilus sp.	México	Mesoamerican Reef, Akumal	20°24'12"N 87°17'56"W	KW591b				MK896698	MK896754		
Dimorphilus gyrociliatus[3]	China	Xiamen, Yellow Sea (wild strain cultured by B. Åkesson)	24°28'47"N 118°05'21"E	KW577a	MK894545	MK894485	MK894489	MK896685	MK896738	+	
Dimorphilus gyrociliatus[3]	China	Xiamen (culture)	24°28'47"N 118°05'21"E	KW577b				MK896686	MK896739		
Dimorphilus gyrociliatus[3]	China	Xiamen (culture)	24°28'47"N 118°05'21"E	KW577c				MK896687	MK896740		
Dimorphilus cf. *gyrociliatus*	Japan	Asamushi Marine Station, Aomori (aquarium)	40°54'11.6"N 140°51'19.4"E	KW741	MK894547	MK894481	MK894488		MK896762	+	

Dimorphilus gyrociliatus	Italy	Anton Dohrn Zoological Station, Napoli (aquarium)	40°51'06"N 14°16'05"E	Ext2877	MK894546	MK894482	MK894487	MK896663	MK896715	+
Dimorphilus gyrociliatus	Italy	Napoli (aquarium)	40°51'06"N 14°16'05"E	KW580a	MK894549	MK894484			MK896744	
Dimorphilus gyrociliatus	Italy	Napoli (aquarium)	40°51'06"N 14°16'05"E	KW580b					MK896745	
Dimorphilus gyrociliatus	Italy	Napoli (aquarium)	40°51'06"N 14°16'05"E	KW580c					MK896746	
Dimorphilus cf. *kincaidi*	United States of America	Scripps Institute of Oceanography (aquarium)	32°51'58.8"N 117°15'12.9"W		MK894548	MK894483	MK894490	MK896714		+
Mesonerilla intermedia	Italy	La Maddalena, Budelli Island, Cavaliere bay, Sardinia	41°17'30"N 09°20'52"E	KW229	MK894550	MK894486		MK896665		+
Diurodrilus subterraneus[4]	Germany	List, Sylt			KC790349	KC790349		KC790350		+
Protodorvillea kefersteinii[5]	France	Roscoff			AF412799	AY732230	AY838843			
Phylo foetida[6]	United States of America	Southern New England, MA			AY532356		AY532337	FJ612511		+

References:

1. Kajihara, H., Ikoma, M., Yamasaki, H. & Hiruta, S. F. *Trilobodrilus itoi* sp. nov., with a re-description of *T. nipponicus* (Annelida: Dinophilidae) and a molecular phylogeny of the genus. *Zool. Sci.* **32,** 405–417 (2015).
2. Kerbl, A., Vereide, E. H., Gonzalez, B. C., Rouse, G. W. & Worsaae, K. Two new meiofaunal species of *Trilobodrilus* (Dinophilidae, Annelida) from California, USA. *Europ. J. Tax.* **421,** 1–18 (2018).
3. Dahlgren, T. G., Åkesson, B., Schander, C., Halanych, K. M. & Sundberg, P. Molecular phylogeny of the model annelid *Ophryotrocha*. *Biol. Bull.* **201,** 193–203 (2001).
4. Golombek, A., Tobergte, S., Nesnidal, M. P., Purschke, G. & Struck, T. H. Mitochondrial genomes to the rescue - Diurodrilidae in the myzostomid trap. *Mol. Phylogenet. Evol.* **68,** 312–326 (2013).
5. Struck, T. H., Halanych, K. M. & Purschke, G. Dinophilidae (Annelida) is most likely not a progenetic Eunicida: Evidence from 18S and 28S rDNA. *Mol. Phylogenet. Evol.* **37,** 619–623 (2005).
6. Bleidorn, C. Phylogenetic relationships and evolution of Orbiniidae (Annelida, Polychaeta) based on molecular data. *Zool. J. Linn. Soc.* **144,** 59–73 (2005).

Supplementary Table 2. Pairwise comparison of distances between COI gene fragments. For accession numbers, see Supplementary Table 1.

			1	2	3	4	5	6	7	8	9	10	11	12	13	14	15	16	17	18	19	20	21	22	23	24	25	26	27	28	29	30	31	32	33	34	35	36	37
**1**	**Nipissat, GL**	KW763																																					
**2**	**Nipissat, GL**	KW764	0,000																																				
**3**	**Nipissat, GL**	KW574b	0,002	0,002																																			
**4**	**Nipissat, GL**	KW574f	0,003	0,003	0,002																																		
**5**	**Ikka Fjord, GL**	KW590a	0,008	0,008	0,010	0,011																																	
**6**	**Ikka Fjord, GL**	KW590b	0,000	0,000	0,002	0,003	0,008																																
**7**	**Ikka Fjord, GL**	KW590c	0,002	0,002	0,003	0,005	0,010	0,002																															
**8**	**Sandavágur, FO**	KW572a	0,003	0,003	0,005	0,006	0,011	0,003	0,005																														
**9**	**Sandavágur, FO**	KW572b	0,000	0,000	0,002	0,003	0,008	0,000	0,002	0,003																													
**10**	**Sandavágur, FO**	KW572c	0,000	0,000	0,002	0,003	0,008	0,000	0,002	0,003	0,000																												
**11**	**Hvalvik, FO**	KW185	0,003	0,003	0,005	0,006	0,011	0,003	0,005	0,006	0,003	0,003																											
**12**	**Áir, FO**	KW573a	0,003	0,003	0,005	0,006	0,011	0,003	0,005	0,006	0,003	0,003	0,006																										
**13**	**Áir, FO**	KW573b	0,002	0,002	0,003	0,005	0,010	0,002	0,003	0,002	0,002	0,002	0,005	0,005																									
**14**	**Áir, FO**	KW573c	0,000	0,000	0,002	0,003	0,008	0,000	0,002	0,003	0,000	0,000	0,003	0,003	0,002																								
**15**	**Sund, FO**	KW581	0,002	0,002	0,003	0,005	0,010	0,002	0,003	0,005	0,002	0,002	0,005	0,005	0,003	0,002																							
**16**	**Sund, FO**	KW582	0,003	0,003	0,005	0,006	0,011	0,003	0,005	0,006	0,003	0,003	0,006	0,006	0,005	0,003	0,005																						
**17**	**Sund, FO**	KW589	0,002	0,002	0,003	0,005	0,010	0,002	0,003	0,005	0,002	0,002	0,005	0,005	0,003	0,002	0,003	0,005																					
**18**	**Porkeri, FO**	KW642a	0,002	0,002	0,003	0,005	0,010	0,002	0,003	0,005	0,002	0,002	0,005	0,005	0,003	0,002	0,003	0,005	0,003																				
**19**	**Porkeri, FO**	KW642b	0,003	0,003	0,005	0,006	0,011	0,003	0,005	0,006	0,003	0,003	0,006	0,006	0,005	0,003	0,005	0,006	0,005	0,005																			
**20**	**Porkeri, FO**	KW642c	0,000	0,000	0,002	0,003	0,008	0,000	0,002	0,003	0,000	0,000	0,003	0,003	0,002	0,000	0,002	0,003	0,002	0,002	0,003																		
**21**	**Borðoyarvík, FO**	KW571a	0,005	0,005	0,006	0,008	0,013	0,005	0,006	0,005	0,005	0,005	0,008	0,008	0,003	0,005	0,006	0,008	0,006	0,006	0,008	0,005																	
**22**	**Borðoyarvík, FO**	KW571b	0,000	0,000	0,002	0,003	0,008	0,000	0,002	0,003	0,000	0,000	0,003	0,003	0,002	0,000	0,002	0,003	0,002	0,002	0,003	0,000	0,005																
**23**	**Borðoyarvík, FO**	KW571c	0,002	0,002	0,003	0,005	0,010	0,002	0,003	0,005	0,002	0,002	0,005	0,005	0,003	0,002	0,003	0,005	0,003	0,003	0,005	0,002	0,006	0,002															
**24**	**Longyearbyen, N**	KW576a	0,011	0,011	0,013	0,015	0,020	0,011	0,013	0,015	0,011	0,011	0,011	0,015	0,013	0,011	0,013	0,015	0,013	0,013	0,015	0,011	0,016	0,011	0,013														
**25**	**Longyearbyen, N**	KW576b	0,011	0,011	0,013	0,015	0,020	0,011	0,013	0,015	0,011	0,011	0,011	0,011	0,013	0,011	0,013	0,015	0,013	0,013	0,015	0,011	0,016	0,011	0,013	0,003													
**26**	**Longyearbyen, N**	KW576c	0,011	0,011	0,013	0,015	0,020	0,011	0,013	0,015	0,011	0,011	0,011	0,015	0,013	0,011	0,013	0,015	0,013	0,013	0,015	0,011	0,016	0,011	0,013	0,003	0,003												
**27**	**White Sea, RU**	KW510	0,010	0,010	0,011	0,013	0,018	0,010	0,011	0,013	0,010	0,010	0,010	0,013	0,011	0,010	0,011	0,013	0,011	0,011	0,013	0,010	0,015	0,010	0,011	0,005	0,005	0,005											
**28**	**White Sea, RU**	KW768	0,010	0,010	0,011	0,013	0,018	0,010	0,011	0,013	0,010	0,010	0,010	0,013	0,011	0,010	0,011	0,013	0,011	0,011	0,013	0,010	0,015	0,010	0,011	0,005	0,005	0,005	0,000										
**29**	**White Sea, RU**	KW769	0,013	0,013	0,015	0,016	0,022	0,013	0,015	0,016	0,013	0,013	0,013	0,013	0,015	0,013	0,015	0,016	0,015	0,015	0,016	0,013	0,018	0,013	0,015	0,008	0,005	0,008	0,003	0,003									
**30**	**White Sea, RU**	KW578a	0,016	0,016	0,018	0,020	0,025	0,016	0,018	0,020	0,016	0,016	0,016	0,020	0,018	0,016	0,018	0,020	0,018	0,018	0,016	0,016	0,022	0,016	0,018	0,011	0,011	0,011	0,006	0,006	0,010								
**31**	**White Sea, RU**	KW578b	0,015	0,015	0,016	0,018	0,023	0,015	0,016	0,018	0,015	0,015	0,015	0,018	0,016	0,015	0,016	0,018	0,016	0,016	0,018	0,015	0,020	0,015	0,016	0,010	0,010	0,010	0,008	0,008	0,011	0,015							
**32**	**White Sea, RU**	KW578c	0,011	0,011	0,013	0,015	0,020	0,011	0,013	0,015	0,011	0,011	0,011	0,015	0,013	0,011	0,013	0,015	0,013	0,013	0,015	0,011	0,016	0,011	0,013	0,006	0,006	0,006	0,002	0,002	0,005	0,008	0,010						
**33**	**Kristineberg, SE**	KW575a	0,013	0,013	0,015	0,016	0,021	0,013	0,015	0,016	0,013	0,013	0,016	0,013	0,015	0,013	0,015	0,016	0,015	0,015	0,013	0,013	0,018	0,013	0,015	0,022	0,018	0,022	0,020	0,020	0,020	0,020	0,025	0,022					
**34**	**Kristineberg, SE**	KW575b	0,016	0,016	0,018	0,020	0,023	0,016	0,018	0,020	0,016	0,016	0,020	0,016	0,018	0,016	0,018	0,020	0,018	0,018	0,016	0,016	0,021	0,016	0,018	0,025	0,022	0,025	0,023	0,023	0,023	0,027	0,028	0,025	0,006				
**35**	**Kristineberg, SE**	KW575c	0,015	0,015	0,016	0,018	0,023	0,015	0,016	0,018	0,015	0,015	0,018	0,015	0,016	0,015	0,016	0,015	0,016	0,016	0,015	0,015	0,020	0,015	0,016	0,023	0,020	0,023	0,022	0,022	0,022	0,025	0,027	0,023	0,005	0,008			
**36**	**Cornwall, UK**	KW756	0,165	0,165	0,168	0,171	0,168	0,165	0,168	0,168	0,165	0,165	0,165	0,160	0,165	0,165	0,168	0,160	0,168	0,168	0,160	0,165	0,170	0,165	0,168	0,168	0,168	0,170	0,170	0,170	0,164	0,170	0,177	0,167	0,160	0,165	0,162		
**37**	**Cornwall, UK**	KW757	0,152	0,152	0,155	0,157	0,155	0,152	0,155	0,154	0,152	0,152	0,152	0,147	0,152	0,152	0,155	0,157	0,155	0,155	0,147	0,152	0,156	0,152	0,154	0,157	0,152	0,154	0,154	0,154	0,148	0,154	0,161	0,151	0,144	0,149	0,152	0,030	
**38**	**Cornwall, UK**	KW758	0,165	0,165	0,168	0,171	0,168	0,165	0,168	0,168	0,165	0,165	0,165	0,160	0,165	0,165	0,168	0,160	0,168	0,168	0,160	0,165	0,170	0,165	0,168	0,168	0,168	0,170	0,170	0,170	0,164	0,170	0,177	0,167	0,160	0,162	0,162	0,003	0,030

Supplementary Table 3. Pairwise comparison of distances between CytB gene fragments. For accession numbers, see Supplementary Table 1.

			1	2	3	4	5	6	7	8	9	10	11	12	13	14	15	16	17	18	19	20	21	22	23	24	25	26	27	28	29	30	31	32	33	34	35	36	37	38	39
1	**Nipissat, GL**	KW763																																							
2	**Nipissat, GL**	KW764	0,005																																						
3	**Nipissat, GL**	KW574a	0,008	0,003																																					
4	**Nipissat, GL**	KW574b	0,008	0,003	0,005																																				
5	**Nipissat, GL**	KW574d	0,005	0,005	0,008	0,008																																			
6	**Nipissat, GL**	KW574f	0,008	0,003	0,005	0,005	0,008																																		
7	**Ikka Fjord, GL**	KW590a	0,005	0,000	0,003	0,003	0,005	0,003																																	
8	**Ikka Fjord, GL**	KW590b	0,003	0,003	0,005	0,005	0,003	0,005	0,003																																
9	**Ikka Fjord, GL**	KW590c	0,008	0,003	0,005	0,005	0,008	0,005	0,003	0,005																															
10	**Sandavágur, FO**	KW572a	0,005	0,000	0,003	0,003	0,005	0,003	0,000	0,003	0,003																														
11	**Sandavágur, FO**	KW572b	0,003	0,003	0,005	0,005	0,003	0,005	0,003	0,000	0,005	0,003																													
12	**Sandavágur, FO**	KW572c	0,008	0,003	0,005	0,005	0,008	0,005	0,003	0,005	0,000	0,003	0,005																												
13	**Hvalvik, FO**	KW185	0,008	0,003	0,005	0,005	0,008	0,005	0,003	0,005	0,005	0,003	0,005	0,005																											
14	**Áir, FO**	KW573a	0,008	0,003	0,005	0,005	0,008	0,005	0,003	0,005	0,000	0,003	0,005	0,000	0,005																										
15	**Áir, FO**	KW573b	0,008	0,003	0,005	0,005	0,008	0,005	0,003	0,005	0,005	0,003	0,005	0,005	0,005	0,005																									
16	**Áir, FO**	KW573c	0,005	0,000	0,003	0,003	0,005	0,003	0,000	0,003	0,003	0,000	0,003	0,003	0,003	0,003	0,003																								
17	**Sund, FO**	KW581	0,008	0,003	0,005	0,005	0,008	0,005	0,003	0,005	0,005	0,003	0,005	0,005	0,005	0,005	0,005	0,003																							
18	**Sund, FO**	KW582	0,010	0,005	0,008	0,008	0,010	0,008	0,005	0,008	0,008	0,005	0,008	0,008	0,008	0,008	0,003	0,005	0,008																						
19	**Sund, FO**	KW589	0,010	0,005	0,008	0,008	0,010	0,008	0,005	0,008	0,008	0,005	0,008	0,008	0,008	0,008	0,008	0,005	0,008	0,010																					
20	**Porkeri, FO**	KW642a	0,005	0,000	0,003	0,003	0,005	0,003	0,000	0,003	0,003	0,000	0,003	0,003	0,003	0,003	0,003	0,000	0,003	0,005	0,005																				
21	**Porkeri, FO**	KW642b	0,008	0,003	0,005	0,005	0,008	0,005	0,003	0,005	0,000	0,003	0,005	0,000	0,005	0,000	0,005	0,003	0,005	0,008	0,008	0,003																			
22	**Porkeri, FO**	KW642c	0,008	0,003	0,005	0,005	0,008	0,005	0,003	0,005	0,005	0,003	0,005	0,005	0,005	0,005	0,005	0,003	0,005	0,008	0,008	0,003	0,005																		
23	**Borðoyarvík, FO**	KW571a	0,008	0,003	0,005	0,005	0,008	0,005	0,003	0,005	0,005	0,003	0,005	0,005	0,005	0,005	0,005	0,003	0,005	0,008	0,008	0,003	0,005	0,005																	
24	**Borðoyarvík, FO**	KW571b	0,008	0,003	0,005	0,005	0,008	0,005	0,003	0,005	0,005	0,003	0,005	0,005	0,005	0,005	0,005	0,003	0,005	0,008	0,008	0,003	0,005	0,005	0,005																
25	**Borðoyarvík, FO**	KW571c	0,016	0,010	0,013	0,013	0,016	0,013	0,010	0,013	0,013	0,010	0,013	0,013	0,013	0,013	0,013	0,010	0,013	0,016	0,016	0,010	0,013	0,008	0,013	0,008															
26	**Longyearbyen, N**	KW576a	0,008	0,003	0,005	0,005	0,008	0,005	0,003	0,005	0,005	0,003	0,005	0,005	0,005	0,005	0,005	0,003	0,005	0,008	0,008	0,003	0,005	0,005	0,005	0,005	0,013														
27	**Longyearbyen, N**	KW576b	0,010	0,005	0,008	0,008	0,010	0,008	0,005	0,008	0,008	0,005	0,008	0,008	0,008	0,008	0,008	0,005	0,008	0,010	0,010	0,005	0,008	0,008	0,008	0,008	0,016	0,003													
28	**Longyearbyen, N**	KW576c	0,010	0,005	0,008	0,008	0,005	0,008	0,005	0,008	0,008	0,005	0,008	0,008	0,008	0,008	0,008	0,005	0,008	0,010	0,010	0,005	0,008	0,008	0,008	0,008	0,016	0,003	0,005												
29	**White Sea, RU**	KW510	0,008	0,003	0,005	0,005	0,008	0,005	0,003	0,005	0,005	0,003	0,005	0,005	0,005	0,005	0,005	0,003	0,005	0,008	0,008	0,003	0,005	0,005	0,005	0,005	0,013	0,000	0,003	0,003											
30	**White Sea, RU**	KW768	0,008	0,003	0,005	0,005	0,008	0,005	0,003	0,005	0,005	0,003	0,005	0,005	0,005	0,005	0,005	0,003	0,005	0,008	0,008	0,003	0,005	0,005	0,005	0,005	0,013	0,000	0,003	0,003	0,000										
31	**White Sea, RU**	KW769	0,010	0,005	0,008	0,008	0,010	0,008	0,005	0,008	0,003	0,005	0,008	0,003	0,008	0,003	0,008	0,005	0,008	0,010	0,010	0,005	0,003	0,008	0,008	0,008	0,016	0,003	0,005	0,005	0,003	0,003									
32	**White Sea, RU**	KW578a	0,008	0,003	0,005	0,005	0,008	0,005	0,003	0,005	0,005	0,003	0,005	0,005	0,005	0,005	0,005	0,003	0,005	0,008	0,008	0,003	0,005	0,005	0,005	0,005	0,013	0,000	0,003	0,003	0,000	0,000	0,003								
33	**White Sea, RU**	KW578b	0,013	0,008	0,010	0,010	0,013	0,010	0,008	0,010	0,010	0,008	0,010	0,010	0,010	0,010	0,010	0,008	0,010	0,013	0,013	0,008	0,010	0,010	0,010	0,005	0,013	0,005	0,008	0,008	0,005	0,005	0,008	0,005							
34	**White Sea, RU**	KW578c	0,010	0,005	0,008	0,008	0,010	0,008	0,005	0,008	0,008	0,005	0,008	0,008	0,008	0,008	0,008	0,005	0,008	0,010	0,010	0,005	0,008	0,008	0,008	0,008	0,016	0,003	0,005	0,005	0,003	0,003	0,005	0,003	0,008						
35	**Kristineberg, SE**	KW575a	0,010	0,005	0,008	0,008	0,010	0,008	0,005	0,008	0,008	0,005	0,008	0,008	0,008	0,008	0,008	0,005	0,008	0,010	0,010	0,005	0,008	0,008	0,008	0,003	0,010	0,003	0,005	0,005	0,003	0,003	0,005	0,003	0,003	0,005					
36	**Kristineberg, SE**	KW575b	0,010	0,010	0,013	0,013	0,010	0,013	0,010	0,008	0,013	0,010	0,008	0,013	0,013	0,013	0,013	0,010	0,013	0,016	0,016	0,010	0,013	0,013	0,013	0,008	0,016	0,008	0,010	0,010	0,008	0,008	0,010	0,008	0,008	0,005	0,005				
37	**Kristineberg, SE**	KW575c	0,005	0,010	0,013	0,013	0,010	0,013	0,010	0,008	0,013	0,010	0,008	0,013	0,013	0,013	0,013	0,010	0,013	0,016	0,016	0,010	0,013	0,013	0,013	0,008	0,016	0,008	0,010	0,010	0,008	0,008	0,010	0,008	0,008	0,010	0,005	0,005			
38	**Cornwall, UK**	KW756	0,150	0,154	0,150	0,158	0,158	0,157	0,154	0,154	0,150	0,154	0,154	0,150	0,158	0,150	0,158	0,154	0,150	0,162	0,146	0,154	0,150	0,150	0,150	0,158	0,162	0,154	0,158	0,158	0,154	0,154	0,150	0,154	0,158	0,154	0,158	0,158	0,154		
39	**Cornwall, UK**	KW757	0,166	0,170	0,166	0,175	0,175	0,174	0,170	0,170	0,166	0,170	0,170	0,166	0,175	0,166	0,175	0,170	0,166	0,170	0,162	0,170	0,166	0,166	0,166	0,175	0,179	0,170	0,175	0,175	0,170	0,170	0,166	0,170	0,175	0,170	0,175	0,175	0,170	0,018	
40	**Cornwall, UK**	KW758	0,168	0,172	0,168	0,176	0,176	0,176	0,172	0,172	0,168	0,172	0,172	0,168	0,176	0,168	0,176	0,172	0,168	0,181	0,164	0,172	0,168	0,168	0,168	0,176	0,181	0,172	0,176	0,176	0,172	0,172	0,168	0,172	0,176	0,172	0,176	0,176	0,172	0,015	0,034

Supplementary Table 4. Pairwise comparison of distances between 16S gene fragments. For accession numbers, see Supplementary Table 1.

			1	2	3	4	5	6	7	8	9	10	11	12	13	14	15	16	17	18
1	**Nipissat, GL**	KW763																		
2	**Nipissat, GL**	KW764	0,000																	
3	**Nipissat, GL**	KW574b	0,000	0,000																
4	**Ikka Fjord, GL**	KW590a	0,003	0,003	0,003															
5	**Sandavágur, FO**	KW572a	0,003	0,003	0,003	0,005														
6	**Hvalvik, FO**	KW185	0,003	0,003	0,003	0,005	0,000													
7	**Áir, FO**	KW573a	0,003	0,003	0,003	0,005	0,000	0,000												
8	**Sund, FO**	KW581a	0,003	0,003	0,003	0,005	0,000	0,000	0,000											
9	**Porkeri, FO**	KW642a	0,003	0,003	0,003	0,005	0,000	0,000	0,000	0,000										
10	**Borðoyarvík, FO**	KW571a	0,003	0,003	0,003	0,005	0,000	0,000	0,000	0,000	0,000									
11	**Longyearbyen, N**	KW576a	0,003	0,003	0,003	0,005	0,003	0,003	0,003	0,003	0,003	0,003								
12	**White Sea, RU**	KW510	0,005	0,005	0,005	0,008	0,005	0,005	0,005	0,005	0,005	0,005	0,003							
13	**White Sea, RU**	KW768	0,003	0,003	0,003	0,005	0,003	0,003	0,003	0,003	0,003	0,003	0,000	0,003						
14	**White Sea, RU**	KW769	0,003	0,003	0,003	0,005	0,003	0,003	0,003	0,003	0,003	0,003	0,000	0,003	0,000					
15	**White Sea, RU**	KW578a	0,003	0,003	0,003	0,005	0,003	0,003	0,003	0,003	0,003	0,003	0,000	0,003	0,000	0,000				
16	**Kristineberg, SE**	KW575a	0,003	0,003	0,003	0,005	0,000	0,000	0,000	0,000	0,000	0,000	0,003	0,005	0,003	0,003	0,003			
17	**Cornwall, UK**	KW756	0,034	0,034	0,034	0,034	0,031	0,031	0,031	0,031	0,031	0,031	0,034	0,037	0,034	0,034	0,034	0,031		
18	**Cornwall, UK**	KW757	0,037	0,037	0,037	0,037	0,034	0,034	0,034	0,034	0,034	0,034	0,037	0,040	0,037	0,037	0,037	0,034	0,008	
19	**Cornwall, UK**	KW758	0,034	0,034	0,034	0,034	0,031	0,031	0,031	0,031	0,031	0,031	0,034	0,037	0,034	0,034	0,034	0,031	0,000	0,008

Supplementary Table 5. Pairwise comparison of distances between 18S gene fragments. For accession numbers, see Supplementary Table 1.

			1	2	3	4	5	6	7	8	9	10	11	12	13	14	15
**1**	**Nipissat, GL**	KW763															
**2**	**Nipissat, GL**	KW764	0,000														
**3**	**Ikka Fjord, GL**	KW590a	0,000	0,000													
**4**	**Sandavágur, FO**	KW572b	0,000	0,000	0,000												
**5**	**Hvalvik, FO**	KW185	0,000	0,000	0,000	0,000											
**6**	**Áir, FO**	KW573a	0,000	0,000	0,000	0,000	0,000										
**7**	**Sund, FO**	KW581	0,000	0,000	0,000	0,000	0,000	0,000									
**8**	**Porkeri, FO**	KW642a	0,000	0,000	0,000	0,000	0,000	0,000	0,000								
**9**	**Borðoyarvík, FO**	KW571a	0,000	0,000	0,000	0,000	0,000	0,000	0,000	0,000							
**10**	**Longyearbyen, N**	KW576a	0,000	0,000	0,000	0,000	0,000	0,000	0,000	0,000	0,000						
**11**	**White Sea, RU**	KW510	0,000	0,000	0,000	0,000	0,000	0,000	0,000	0,000	0,000	0,000					
**12**	**White Sea, RU**	KW768	0,000	0,000	0,000	0,000	0,000	0,000	0,000	0,000	0,000	0,000	0,000				
**13**	**White Sea, RU**	KW769	0,000	0,000	0,000	0,000	0,000	0,000	0,000	0,000	0,000	0,000	0,000	0,000			
**14**	**White Sea, RU**	KW578a	0,000	0,000	0,000	0,000	0,000	0,000	0,000	0,000	0,000	0,000	0,000	0,000	0,000		
**15**	**Kristineberg, SE**	KW575a	0,000	0,000	0,000	0,000	0,000	0,000	0,000	0,000	0,000	0,000	0,000	0,000	0,000	0,000	
**16**	**Cornwall, UK**	KW757	0,001	0,001	0,001	0,001	0,001	0,001	0,001	0,001	0,001	0,001	0,001	0,001	0,001	0,001	0,001

Supplementary Table 6. Pairwise comparison of distances between 28S gene fragments. For accession numbers, see Supplementary Table 1.

			1	2	3	4	5	6	7	8	9	10	11	12	13
**1**	**Nipissat, GL**	KW763													
**2**	**Ikka Fjord, GL**	KW590a	0,002												
**3**	**Sandavágur, FO**	KW572a	0,003	0,001											
**4**	**Hvalvik, FO**	KW185	0,001	0,003	0,004										
**5**	**Áir, FO**	KW573a	0,002	0,000	0,001	0,003									
**6**	**Sund, FO**	KW581	0,002	0,000	0,001	0,003	0,000								
**7**	**Porkeri, FO**	KW642a	0,002	0,003	0,004	0,003	0,003	0,003							
**8**	**Borðoyarvík, FO**	KW571a	0,003	0,001	0,002	0,004	0,001	0,001	0,004						
**9**	**Longyearbyen, N**	KW576a	0,002	0,003	0,004	0,003	0,003	0,003	0,000	0,004					
**10**	**White Sea, RU**	KW510	0,000	0,002	0,003	0,001	0,002	0,002	0,002	0,003	0,002				
**11**	**White Sea, RU**	KW768	0,000	0,002	0,003	0,001	0,002	0,002	0,002	0,003	0,002	0,000			
**12**	**White Sea, RU**	KW578a	0,004	0,002	0,003	0,005	0,002	0,002	0,003	0,003	0,003	0,004	0,004		
**13**	**Kristineberg, SE**	KW575a	0,003	0,001	0,002	0,004	0,001	0,001	0,004	0,002	0,004	0,003	0,003	0,001	
**14**	**Cornwall, UK**	KW757	0,003	0,001	0,002	0,004	0,001	0,001	0,004	0,002	0,004	0,003	0,003	0,003	0,002
